# Gestational weight gain according to treatment in gestational diabetes: a systematic review and meta-analysis

**DOI:** 10.61622/rbgo/2025rbgo65

**Published:** 2025-09-08

**Authors:** Carolina de Freitas Alves Amaral-Moreira, Daiane Sofia de Morais Paulino, José Paulo Siqueira Guida, Belmiro Gonçalves Pereira, Patrícia Moretti Rehder, Fernanda Garanhani Surita

**Affiliations:** 1 Universidade Estadual de Campinas Faculdade de Ciências Médicas Campinas SP Brazil Faculdade de Ciências Médicas, Universidade Estadual de Campinas, Campinas, SP Brazil.

**Keywords:** Gestational diabetes, Gestational weight gain, Metformin, Insulin

## Abstract

**Objective:**

In this systematic review, we aim to compare the GWG in pregnant women with diabetes treated with metformin and other interventions

**Methods:**

Data Sources: The searched baselines included PubMed, Scopus, Web of Science, Embase, and Virtual Health Library (BVS). Study selection: We selected articles that compared the GWG in women with diabetes treated with metformin or insulin. We have included clinical trials (randomized or not), observational studies (cohort, case control, and cross-sectional). Reviews (systematic or not), posters, event abstracts, and letters were excluded. Data Collection: We pooled odds ratios (OR) and mean difference (MD) and used a random effect model using R Studio software to compare the weight gain, fetal birthweight and preeclampsia according to treatment.

**Results:**

On research conducted in January 2024, with no data limit of the search, 433 trials were identified, of which 175 remained after duplicate removal. 50 studies were analyzed in the full text analyses and 9 were selected for the systematic review. 8 studies demonstrated that gestational weight gain during metformin treatment is lower when compared to other treatments, especially insulin, although it was not different from other outcomes. Meta-analyses demonstrated that oral medication GWG is lower than insulin with a standard mean difference (SMD) -1,05 [-1,87, - 0,23].

**Conclusion:**

Oral medication has a lower gestational weight gain in patients with gestational diabetes when compared to insulin.

**International Prospective register of Systematic Reviews (PROSPERO):**

CRD 42024492158

## Introduction

Excessive gestational weight gain (GWG) is one of the main concerns in antenatal care, since it is associated with hypertensive diseases, cesarean section, large for gestational age (LGA) newborns, and increases the risk for future type 2 diabetes in the mothers and childhood obesity in the offspring.^(1)^ It is also common in women diagnosed with gestational diabetes (GDM) due to the pathophysiology of both conditions being associated with insulin resistance, especially in women who are already obese.^(1–4)^

Diabetes in pregnancy is an independent risk factor for adverse maternal and neonatal outcomes, and for maternal health, especially if glycemic levels are inadequate. Newborns of diabetic women have a higher risk of neonatal hypoglycemia, LGA, respiratory distress, preterm birth and a higher risk of metabolic diseases as the mother are at a higher risk of cesarean section and preeclampsia as increases the risk for cardiovascular diseases.(^4,5^)

Lifestyle changes are the first line of treatment; however, some women require drug intervention.^(1)^ Insulin is the drug of choice for GDM, and it is a safe medication for the fetus, although it seems to be associated with an increase in GWG.^(6)^ Metformin is an alternative to provide proper glycemic control in pregnancy as it is easier to take.^(7)^ There are concerns regarding the crossing of the placental barrier; however, evidence suggests that it does not increase congenital anomalies. The treatment with metformin seems to have a lower incidence of large for gestational age and macrosomia, when compared to insulin.^(7–9)^ Also, metformin seems to have a lower GWG, which is an advantage for patients with diabetes and obesity, a common association known as diabesity.^(7,10)^

In non-pregnant population, the first line of treatment is metformin since it acts in the pathophysiology of diabetes, the insulin resistance, and reduces diabetes progression to diabetes type 2 in patients with impaired glucose tolerance (IGT).^(11)^ It is also well established that weight loss is effective in remission IGT and diabetes, therefore is an important goal in the treatment.^(12)^

Since excessive GWG, a potential consequence of uncontrolled GDM, is an independent risk for cesarean section, LGA, and hypertensive disorders metformin seems to be an interesting alternative for GDM treatment, especially in obese and overweight women. This study aimed to investigate the GWG among pregnant women exposed to metformin or insulin.^(13)^

## Methods

### Eligibility criteria for studies

We followed the Preferred Reporting Items for Systematic Reviews and Meta-Analyses (PRISMA) guidelines for this systematic review and meta-analysis. This study was previously registered in the *International Prospective register of Systematic Reviews (*PROSPERO*)* under CRD 42024492158.^(14)^

To conduct the research, we formulated a structured question using the acronym PICO(population, intervention, comparison, and outcome):

- *Population:* Pregnant women diagnosed with gestational diabetes or diabetes type II that require drug treatments;
- *Intervention:* The intervention evaluated in this systematic review was the use of metformin in the treatment of gestational diabetes or diabetes type 2 diabetes;
- *Control:* The control was insulin, currently the gold standard for diabetes treatment in pregnancy among other treatments for diabetes;
- *Outcomes:* The primary outcome evaluated in this study was GWG. Other secondary outcomes were the incidence of hypertensive disorders, birth weight, and gestational age at birth.

We have included clinical trials (randomized or not), observational studies (cohort, case control, and cross-sectional). Reviews (systematic or not), posters, events abstracts, and letters were excluded. Only published articles were included, as preprints were excluded. Studies in English, Spanish, or Portuguese were considered.

### Information sources

The databases consulted were PubMed, Scopus, Web of Science, Embase, and BVS.

### Search strategy

The search was conducted in January 2024. The strategy included the terms "gestational diabetes" and "metformin" and "insulin" and "gestational weigh gain". We also included similar terms using the MeSH, Emtree vocabulary for Web of Science and DeCs at Virtual Health Library (BVS). For "gestational diabetes" similar terms were ""Diabetes, Gestational" OR "Pregnancy-Induced Diabetes" " OR "Gestational Diabetes Mellitus" in MeSH and "Diabetes, Gestational" OR "Pregnancy-Induced Diabetes" " OR "Gestational Diabetes Mellitus in Emtree. For "gestational weight gain", MeSH similar terms used were "Pregnancy Weight Gain" OR "Maternal Weight Gain". The consulted databases were PubMed, Scopus, Web of Science, Embase, and BVS. The search strategy is available in the supplemental file). This research was assisted by the Library of the Medical Sciences College of the University of Campinas, which has a specialized service support for systematic reviews.

### Study selection

The articles’ abstracts were exported to the Rayyan® platform for deduplication and screening. At first, studies were screened by title and abstracts, and after the first selection by complete text analysis. Two independent reviewers, CFAAM and DSMP, screened records, and a third reviewer, FGS, resolved conflicts.

## Data extraction

The following data was extracted from each included study: authors, title, country of origin, year of publication, study design, population evaluated, number of patients in each arm of treatment, pre pregnancy body mass index (BMI) or weight before pregnancy, the treatment for diabetes in the study, GWG, birthweight, gestational age at birth, incidence of hypertensive disorders.

### Assessment of risk of bias

Bias assessment of the included studies was conducted individually by the two authors using the "Cochrane Risk of Bias" tool for randomized trials (Rob). The RoB tool assessed the following topics:

- Random sequence generation (selection bias);
- Allocation Concealment (selection bias);
- Selective reporting (reporting bias);
- Blinding participants and personnel (performance bias);
- Blinding outcome assessment (detection bias);
- Incomplete outcome data.

Discrepancies were resolved through consensus. We also used the Grading of Recommendations Assessment and Development and evaluations (GRADE) for the classification of the clinical evidence, which reduces the level of high-quality evidence of randomized controlled trials according to the quality of the original studies, inconsistencies of the results, indirect evidence, imprecision, and publication bias.

### Data Synthesis and Measure of effect

We have performed a qualitative analysis of the included studies, as described below. The included information from the articles were title, year, country, study design, inclusion criteria, number of participants, mean age, mean pre-pregnancy BMI, mean GWG, mean fetal weight, prevalence of preeclampsia and cesarean section. For the meta-analysis, we used a random-effect model for eligible studies. We estimated the effects using R Studio (version 4.3). Forest Plot graphs were generated for each outcome.

## Results

### Study selection

**Figure 1:**
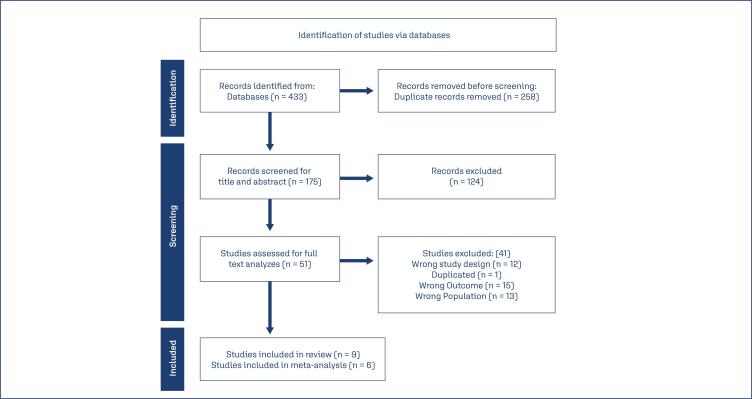
Flow Diagram of included studies

**Source:** Page et al. (2021)^(14)^

The database search identified 433 articles, of which 258 were duplicated. 175 studies were evaluated based on abstract and title; among those, 50 were selected for full text analyzes. Forty-one studies were excluded since 12 studies had a wrong study design, 1 study was duplicated, 15 studies had the wrong outcome, and 13 studies analyzed populations different then pregnant women with diabetes nine studies were included in the review and 6 on the meta-analysis (Figure 1).

This work is licensed under CC BY 4.0. To view a copy of this license, visit https://creativecommons.org/licenses/by/4.0/

### Study characteristics

Of the included studies, seven were randomized controlled trials and two were cohorts. Eight evaluated women with gestational diabetes, while one study evaluated pregnant women with diabetes mellitus type II. Seven studies compared metformin with insulin, one compared insulin, metformin, and glyburide, and one study compared metformin with glyburide. The studies included a total of 2678 women. Studies were performed from 2010 to 2022, in 8 different countries. The summary of the results is presented in chart 1.

**Chart 1:** Articles selected and results

Author	Title	Country	Study design	Inclusion	Analyzed groups	GWG	Other Outcomes
Ainuddin et al. (2015)^(15)^	Metformin treatment in type 2 diabetes in pregnancy: An active controlled, parallel-group, randomized, open label study in patients with type 2 diabetes in pregnancy	Pakistan	RCT	DM2	metformin +- insulin X insulin	GWG was lower in the metformin group	Metformin group had lower rates of pregnancy induced hypertension and hypoglycemia, but had higher rates of small for gestational age
Ainuddin et al. (2015^)(16)^	Metformin versus insulin treatment in gestational diabetes in pregnancy in a developing country. A randomized control trial.	Pakistan	RCT	GDM	metformin +- insulin X insulin	GWG was lower in the metformin group	Metformin group had lower rates or preeclampsia, neonatal morbidity and lower mean birth weight
Galal et al. (2019)^(17)^	Metformin versus insulin in treatment of gestational diabetes mellitus: A randomized controlled trial.	Egypt	RCT	GDM	metformin x insulin	GWG was lower in the metformin group	Metformin was cost effectiveness. Metformin had lower rates of hypoglycemia
Bettencourt-Silva et al. (2019)^(18)^	Metformin in overweight and obese women with gestational diabetes: a propensity score-matched study	Portugal	Cohort	GDM and overweight or obesity	metformin x insulin	Metformin had lower rates of excessive GWG and more adequate GWG	There was no difference in the other outcomes
Huhtala et al. (2020)^(19)^	Metformin and insulin treatment of gestational diabetes: Effects on inflammatory markers and IGF-binding protein-1 – Secondary analysis of a randomized controlled trial.	Finland	RCT	GDM	Metformin X insulin	GWG was lower in the metformin group	Metformin group had smaller babies
Niromanesh et al. (2012)^(20)^	Metformin compared with insulin in the management of gestational diabetes mellitus: a randomized clinical trial	Iran	RCT	GDM	Metformin X insulin	GWG was lower in the metformin group	Metformin group had a higher incidence of smaller babies.
Roy et al. (2018)^(21)^	The use of metformin versus insulin in the management of diabetes mellitus in pregnancy . a randomized control tria	India	RCT	GDM	metformin +- insulin X insulin	GWG was lower in the metformin group	No difference in other outcomes
Silva et al. (2010)^(22)^	Metformin compared with glyburide for the management of gestational diabetes	Brazil	RCT	GDM	metformin +- insulin X glyburide	GWG was lower in the metformin group	No difference in other outcomes
Hay et al. (2022)^(23)^	Prescribing patterns and outcomes among patients treated for gestational diabetes mellitus.	USA	Cohort	GDM	metformin x insulin xgliburide	There was no difference in the GWG between groups	Other outcomes were similar between groups

The main population analyzed included women with GDM, a mean age of 30-35 years old, and a mean BMI of 30 kg/m^2^. Metformin treatment was also similar in the studies, starting with 500 mg/day and up to 2000-2500 mg/day. The study designs, cohort, or RCT were also similar, since they compared different treatment groups, as described in chart 2.

**Chart 2:** Population characteristics

Author	Title	participants	Metformin	Met+ insulin	insulin	glyburide	Mean age	Mean BMI
Ainuddin et al. (2015)^(15)^	Metformin treatment in type 2 diabetes in pregnancy: An active controlled, parallel-group, randomized, open label study in patients with type 2 diabetes in pregnancy	206	16	90	100	0	31	28-35 kg/m²
Ainuddin et al. (2015^)(16)^	Metformin versus insulin treatment in gestational diabetes in pregnancy in a developing country. A randomized control trial.	150	43	32	75	0	30-31	66-67kg
Galal et al. (2019)^(17)^	Metformin versus insulin in treatment of gestational diabetes mellitus: A randomized controlled trial.	106	52	0	54	0	32	30 kg/m²
Bettencourt-Silva et al. (2019)^(18)^	Metformin in overweight and obese women with gestational diabetes: a propensity score-matched study	457	0	177	280	0	34	78-86kg/30-32 kg/m²
Huntala et al. (2020)^(19)^	Metformin and insulin treatment o fgestational diabetes: Effects on inflammatory markers and IGF-binding protein-1 - Secondary analysis of a randomized controlled trial.	227	43	32	75	0	30-31	66-70 kg
Niromanesh et al. (2012)^(20)^	Metformin compared with insulin in the management of gestational diabetes mellitus: a randomized clinical trial	172	80	0	80	0	30-32	27-28 kg/m²
Roy et al. (2018)^(21)^	The use of metformin versus insulin in the management of diabetes mellitus in pregnancy . a randomized control tria	160	80	0	80	0	30-31	26kg/m²
Silva et al. (2010)^(22)^	Metformin compared with glyburide for the management of gestational diabetes	72	40	0	0	32	31-33	28-30 kg/m²
Hay et al. (2022)^(23)^	Prescribing patterns and outcomes among patients treated for gestational diabetes mellitus.	368	50	0	13	283	31	91-98 kg

### Risk of bias on included studies

**Figure 2:**
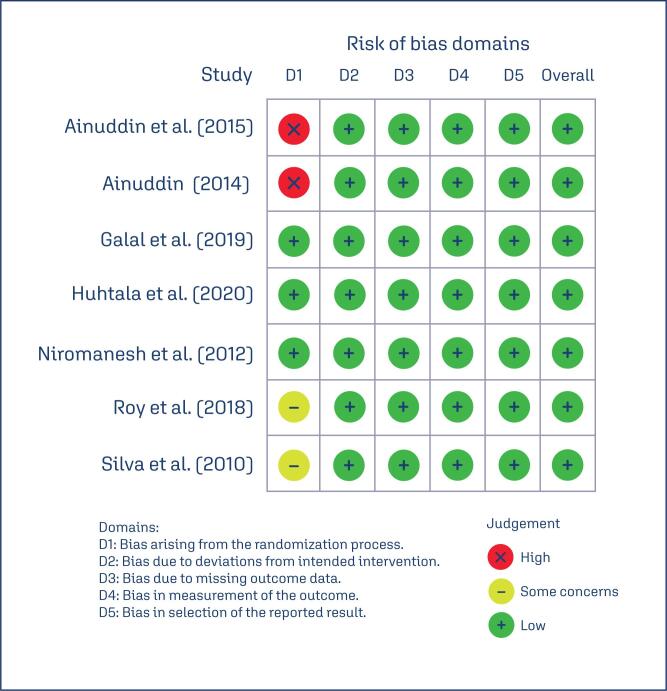
RoB 2 for clinical trials

The Risk of bias using the RoB2 tool for the clinical trials is the following (Figure 2).

All studies were open label, since the drug intake is different from insulin and metformin. As the results are objective measures, we considered that there was a low risk of bias overall.

### Synthesis of results

Of the nine included studies, 8 studies concluded that women treated with metformin had a lower GWG when compared to other treatments.^(15–22)^ The other study showed that the difference was similar between metformin and insulin, compared to glyburide, and the groups had significantly different sizes (metformin 50 patients, insulin 13, and glyburide 283), which might have compromised the statistical evaluation.^(23)^

Among other results, most of them suggested that metformin had advantages in cost effectiveness and less neonatal and maternal morbidity, especially hypoglycemia, than insulin.^(15–17)^ Two studies concluded that metformin had a lower incidence of hypertension and preeclampsia.^(15,16)^ Four studies concluded that babies exposed to metformin were smaller for gestational ages and one study found that there were more preterm births.^(15,17,19,20)^

**Figure 3:**
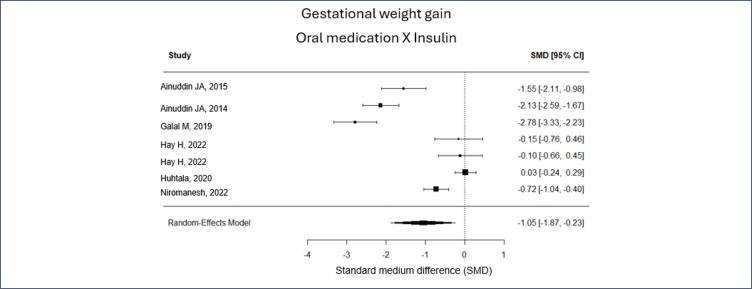
Gestational weight gain according to, oral medication X Insulin

Six studies were included in the meta-analysis. For the first analysis, we analyzed both oral medications (metformin and glyburide), gestational weight gain with insulin, and it was statistically significant, demonstrating that oral medication gestational weight gain is lower than insulin with a standard mean difference (SMD) -1,05 [-1,87, - 0,23]. Mean GWG in the metformin group was from 4 kg to 11,3 Kg while in the insulin group the variation was between 5,4 Kg to 13,7 kg (Figure 3).

**Figure 4:**
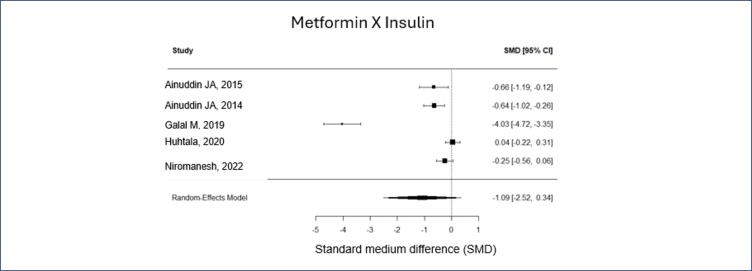
Fetal weight according to, metformin X insulin

**Figure 5:**
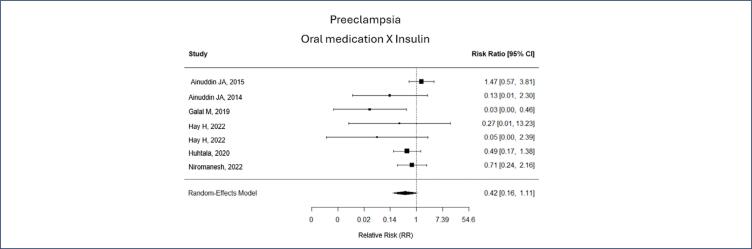
Preeclampsia according to, oral medication X Insulin

For the other outcomes, preeclampsia (PE) and fetal birth weight, the difference was not significant. However, the relative risk tends to be smaller in both analyzes (relative risk of PE 0,42 [0,16-1,11] and SMD for fetal weight of -1,09 [-2,25, 0,34]). Fetal weight variation in the metformin group was from 2,99 kg to 3,61 Kg, while in the insulin group was from 3,22 to 3,7 Kg (Figure 4 and 5).

## Discussion

In the past decade, there was an increase in articles comparing metformin and glyburide with insulin in the treatment of gestational diabetes and type 2 diabetes. Many aspects have been explored in literature like incidence of hypertensive syndromes and efficacy in glycemic controlled, as long term outcomes, However, few studies evaluated the GWG in pregnancy as a first outcome, most of them, like the ones included in this systematic review and meta-analysis, describe the GWG as a secondary outcome. The association of GWG and diabetes is a poorly explored subject in literature. It is established that obesity and excessive GWG are associated with stillbirth, preeclampsia, diabetes post-partum hemorrhage. Therefore, it has been hypothesized if restricting GWG in obese patients would improve pregnancy outcomes.^(24)^

The recommendations of GWG most used worldwide are provided by The Institute of Medicine (IOM) which stipulates the GWG according to pre-pregnancy BMI, and it was established in 2010, without further updates. This guideline has been controversial in patients with obesity, since it stipulates a similar GWG for any grade of obesity, which led to the development of new studies that have demonstrated that lower limits of the proposed by IOM and weight loss during pregnancy decreased the risk of adverse effects, without compromising fetal development.^(24,25)^

Since obesity and excessive GWG are associated with diabetes due insulin resistance, weight control and restricting GWG are interesting goals, in prenatal care of women with gestational diabetes. In this context, as demonstrated by this review, metformin seems an interesting alternative to diabetes treatment in pregnancy and provide better outcomes in this population.

## Conclusion

Considering the high incidence of gestational diabetes associated with obesity, metformin presents itself as a viable alternative and safe alternative for the fetus, with a lower GWG than insulin, and should be considered for treatment, especially when GWG is a concern, as in patients with obesity. Also as the studies suggest, metformin has a lower fetal morbidity, especially with less fetal hypoglycemia.
